# Lgr5 is a potential marker of colorectal carcinoma stem cells that correlates with patient survival

**DOI:** 10.1186/1477-7819-10-244

**Published:** 2012-11-15

**Authors:** Xiao-Song Wu, Hong-Qing Xi, Lin Chen

**Affiliations:** 1 Department of General Surgery, Chinese People’s Liberation Army General Hospital, 28 Fuxing Road, Beijing, 100853, China

**Keywords:** Colorectal cancer, Lgr5, Prognosis, Cancer stem cells

## Abstract

**Background:**

Lgr5 (leucine-rich-repeat-containing G-protein-coupled receptor 5) has recently been identified as an intestinal stem cell marker. In order to determine whether Lgr5 is a potential marker of cancer stem cells, we investigated whether Lgr5 expression correlated with Ki-67 expression and prognosis in colorectal carcinoma.

**Methods:**

Lgr5 and Ki-67 expression were evaluated by immunohistochemistry in 192 colorectal carcinoma specimens. Selection of side population (SP) cells was performed by staining with Hoechest 33342, and Lgr5 expression in Colo205 SP cells was then detected by immunofluorescence.

**Results:**

Lgr5 expression was significantly higher in carcinoma than in normal mucosa (*P*=0.001). Lgr5 was positively correlated with histological grade (*P*=0.001), depth of invasion (*P*=0.001), lymph node metastasis (*P*=0.001), distant metastasis (*P*=0.004), pTNM stage (*P*=0.001), and Ki-67 (r=0.446, *P*=0.001). Multivariate analysis showed that the effect of Lgr5 on survival was independent of Ki-67 (*P*=0.037). In the *in vitro* study, Hoechst low-staining cells were counted in 7% of the Colo205 colon cancer cell line population, and Lgr5 expression was strikingly stronger in Hoechst low-staining cells than in high-staining cells (*P*=0.001).

**Conclusions:**

These findings suggest that Lgr5 may play an important role in the progression and prognosis of colorectal carcinoma, and may be a potential new therapeutic target for the treatment of colorectal cancer patients. It may also be considered as a potential marker for colorectal cancer stem cells (CSCs).

## Background

Tumor progression may be related to the alteration of genes that regulate stem cell renewal. Patients who experience distant metastasis usually have a shorter survival time after diagnosis. Although cancer tumors are targeted as homogenous tissues by many therapies, recent evidence suggests that considering the cancerous tissue to be composed of heterogeneous cells, including cancer stem cells (CSCs), may be more effective. It has been suggested that tumors are generated and maintained by a small subset of cancer cells capable of multi-differentiation and self-renewal, which are known as CSCs [1]. A side population (SP) is a subpopulation of cells that is distinct from the main population. SP cells may exhibit stem cell-like characteristics. Previous studies show that there is increasing evidence for a small SP of cancer stem cells, both from established cancer cell lines and primary tumors [2,3]. This evidence may support a new model of tumorigenesis that involves a subpopulation of stem cells in the cancer and also the regulation of stem cell self-renewal.

Colorectal cancer is the most common malignancy of the gastrointestinal tract [4] and causes 655,000 deaths worldwide every year [5]. Many treatment protocols have been applied to colorectal cancer, but they have not resulted in a complete cure. This may be due to colorectal cancer stem cells (CSCs) that are resistance to radiation therapy and chemotherapy, and may enable the recurrence of cancers. Therefore, it is important to use therapies that target not only proliferating cells but also stem cells in order to cure cancer [6]. Therefore, in order to seek out and eliminate colon CSCs, a specific biomarker is needed. Becker *et al.* suggested that leucine-rich repeat-containing G-protein-coupled receptor 5 (Lgr5) may be a better marker for CSCs in colorectal cancer [7].

Lgr5, which is also known as GPR49, is a member of the G-protein-coupled receptor (GPCR) family of proteins and is a target of Wnt signaling [8-10]. Barker *et al.* recently reported that Lgr5 is a marker of murine small intestine and colon stem cells [11]. Previous studies demonstrated that Lgr5 is overexpressed in hepatocellular carcinoma [10], colorectal cancer [12,13], ovarian cancer [13], basal cell carcinoma [14], and esophageal adenocarcinoma [15]. Recently, it was reported that adenomatous polyposis coli (APC) mutations exclusively in Lgr5-positive cells could promote adenomatous growth in the colon of mice [16]. These data suggested that Lgr5 may play an important role in tumorigenesis. Lgr5 has also been detected in tumor spheres derived from colon cancer [17]. Many authors have suggested that Lgr5 could serve as an ideal marker of colorectal CSCs [18,19]. An adult stem cell subpopulation, termed the ‘side population’ (SP), has been identified that can rapidly efflux the fluorescent dye, Hoechst 33342. SP cells have been defined by Hoechst 33342 staining in many mammals, including humans [20-22]. Ki-67, which is a nuclear nonhistone protein, is a recognized nuclear antigen-specific marker that is used to evaluate the proliferative activities of various tumors. However, to our knowledge, the relationship between the expression of Lgr5 and the expression of Ki-67 in colorectal carcinoma has not yet been investigated. In this study, we investigated the possible role of Lgr5 expression in clinicopathology and prognosis, as well as the relationship between Lgr5 and Ki-67 in colorectal carcinoma. To achieve this, we selected SP cancer stem cells by Hoechst 33342 extrusion and used immunocytochemistry to explore the expression of Lgr5 in Hoechst33342 low-staining cancer cells in the colon cancer cell line, Colo205. The differential expression of Lgr5 between Hoechst 33342 low-staining cells and high-staining cells in colon cancer was observed and analyzed microscopically, and provided useful information for the clinical diagnosis and treatment of CSCs.

## Methods

### Patients and specimens

This retrospective study consisted of 192 colorectal adenocarcinomas with available histopathological data. Patients were diagnosed and treated in our institute from January 2001 to December 2004. The 80 distal normal colorectal tissues were randomly selected from the 192 cases of colorectal cancer as normal controls. Ethical approval for this study was not required by our institution as the experiments carried out did not relate to patients’ privacy, impairment, or treatment. The ages of the patients ranged from 22 to 83 years (median, 62 years; mean, 58.1 years). Of the patients, 120 were men and 72 were women. According to histological grading, 22 patients were at grade 1, 107 were at grade 2, and 63 were at grade 3. According to the clinical TNM stage revised by the International Union Against Cancer (UICC) in 2009, 47 patients were stage I, 70 patients were stage II, 65 patients were stage III, and 10 patients were stage IV. All patients were followed up for survival. By April 2011 (the time of data analysis), 116 patients had died and 76 patients were alive. The median survival time was 59 months.

### Cell line and cell culture

The human colon cancer cell line, Colo205 (ATCC, Manassas, VA, USA), was cultured in RPMI 1640 medium (GIBCO-BRL, Gaithesberg, MD) containing 10% FBS (GIBCO-BRL, Gaithesberg, MD, USA) at 37°C in a humidified 5% CO2/95% air atmosphere.

### Immunohistochemical analysis

Immunohistochemical staining of Lgr5 and Ki-67 was carried out as previously described [23]. Sections (4 μM thick) were cut from paraffin blocks and mounted onto APES-coated glass slides. The sections were deparaffinized in xylene and dehydrated in a graded series of ethanol. Antigen retrieval was performed by heating in 0.01 M citrate buffer (pH 6.0) in a microwave oven for 2 min at 100°C. The slides were then immersed in 3% hydrogen peroxidase-methanol to inhibit endogenous peroxidase activity. After washing with phosphate-buffered saline (PBS), the slides were incubated with primary monoclonal rabbit antibody to human Lgr5 (Abcam, Cambridge, MA, USA) diluted 1:50 in blocking solution, and mouse monoclonal antibody to human Ki-67 (Zymed Laboratories, San Francisco, CA, USA) diluted 1:150 in blocking solution, at 4°C overnight. The sections were then washed in PBS and incubated with Polyperoxidase-anti-mouse/rabbit IgG (Zymed Laboratories, San Francisco, CA, USA) for 20 min. After washing with PBS, 3,3′-Diaminobenzidine was used as the chromogen. Finally, the sections were counterstained with hematoxylin. As a negative control, the primary antibody was replaced with normal rabbit serum.

### Evaluation of score

All specimens were examined by two pathologists who did not possess knowledge of the clinical data. In case of discrepancies, a final score was established by re-assessment on a double-headed microscope. In scoring Lgr5 and Ki-67 expression, both the extent and intensity of the immunopositivity were considered, according to Zhao *et al*. [23], Hao *et al.*[24], and Fan *et al.*[25]. The staining intensity was scored as follows: 0, negative; 1, weak; 2, moderate; 3, strong. The positivity was quantified according to the percentage of positive tumor cells: 0, <5%; 1, >5-25%; 2, >25-50%; 3, >50-75%; 4, >75%. The final score was determined by multiplying the intensity and the quantity scores, which yielded a range from 0 to 12. The expression of Lgr5 and Ki-67 were regarded as positive when the score was >5.

### Microscopoic analysis for Hoechst 33342 extrusion

Cells at a concentration of 106 cells/mL were stained at 37°C for 90 min with 5 μg/mL Hoechst 33342 (Sigma, MO, USA) in 4 mL of Dulbecco’s modified Eagle’s medium containing 2% bovine serum albumin (BSA). Verapamil (Sigma, MO, USA) was also added to a parallel set of samples for 10 min before Hoechst staining to analyze the effect of inhibiting Hoechst extrusion [26,27]. After incubation, the cells were washed with Hanks’ balanced salt solution (Invitrogen Corporation, Grand Island, NY, USA) and fixed with 10% formalin for 120 min. Smear slides were made with Auto Smear CF-120 (SAKURA). The cells with Hoechst 33342 extrusion, which were presented with a low blue fluorescence signal or were negatively stained, were analyzed and recorded using a BX51 fluorescent microscope (Olympus) with a cell counter. Each experiment was performed in triplicate and was repeated at least three times.

### Fluorescent immunocytochemistry

The cells were immersed in goat serum at room temperature for 15 min to block non-specific binding sites. The slides were then incubated with a primary antibody to Lgr5 (Abcam, Cambridge, MA, USA) overnight at 4°C. The cells were then washed with PBS and then incubated with a goat anti-rabbit TRITC conjugated secondary antibody (1:50 dilution; Zymed Laboratories, San Francisco, CA, USA) for 2 h at room temperature. Images were randomly taken at 40×10 magnification with a digital color camera, and the Hoechst 33342 pre-stained cells with positive or negative signals from 10 images containing at least 800 cells were counted and analyzed with image analysis software.

### Statistical analysis

SPSS V.13.0 (SPSS, Chicago, IL, USA) was used for statistical analysis. The Pearson Chi’s square test was used to examine the various clinicopathological characteristics of Lgr5 and Ki-67 expression. The Spearman’s correlation coefficient test was used to assess the relationship between Lgr5 and Ki-67 expression. Univariate survival analysis was conducted according to the Kaplan-Meier method, and the difference between the survival curves was analyzed with the log-rank test. Multivariate survival analysis was performed using the Cox proportional hazard model. Statistical significance was considered at a value of *P* <0.05.

## Results

### Expression of Lgr5 in colorectal carcinoma

**Figure 1:**
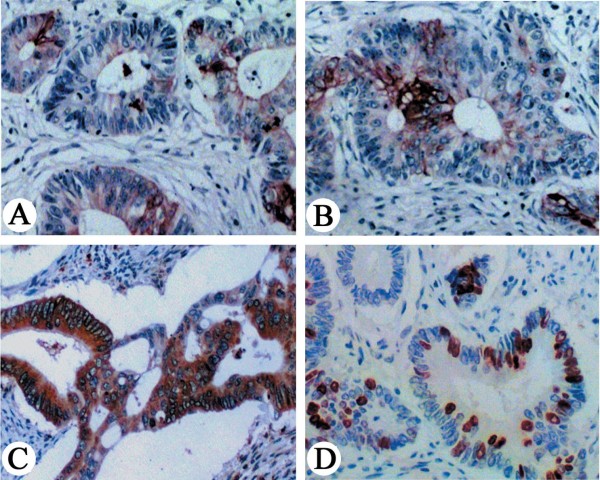
**An example of immunohistochemical staining for Lgr5 and Ki-67 in colorectal carcinoma tissue.** Positive expression of Lgr5 in the cytoplasm of cancer cells. (**A**) A few scattered cells displaying Lgr5 staining. Lgr5-positive cells exhibiting focal (**B**) and patchy (**C**) distribution patterns in some cases of colorectal carcinoma. (**D**) Ki-67 protein was found in the nucleus of cancer cells. (A-D: ×200).

Lgr5 protein was positively expressed in 56.3% (108/192) of the colorectal carcinomas, and 25% (15/60) of distal normal mucosa. Expression of Lgr5 protein was found mostly in the cytoplasm of cancer cells, with some membrane staining (Figure 1A, B, and C). A very significant difference in Lgr5 expression was found between colorectal carcinoma and normal mucosa (*P*=0.001).

### The association between Lgr5 expression and clinicopathological features

Significant positive correlations were found between Lgr5 expression and histological grade (*P*=0.001), depth of invasion (*P*=0.001), lymph node metastasis (*P*=0.001), and distant metastasis (*P*=0.004). Lgr5 expression gradually increased as the pTNM stage progressed (*P*=0.001) (Table 1).

**Table 1:** Relationship of Lgr5 and Ki-67 expression to clinicopathological variables in colorectal cancer

**Variables**	**Lgr5**		***P *value**	**Ki-67**		***P *value**
	**+**	**-**		**+**	**-**	
*Gender*						
Male	66	54	*P*=0.652	71	49	*P*=0.940
Female	42	30		43	29	
*Age (years)*						
<45	18	11	*P*=0.791	19	10	*P*=0.707
45-60	32	26		35	23	
≥60	58	47		60	45	
*Tumor size (cm)*						
d<5	67	61	*P*=0.282	66	62	*P*=0.006
5≤d<10	37	20		42	15	
d≥10	4	3		6	1	
*Histological grade*						
1	6	16	*P*=0.001	10	12	*P*=0.001
2	52	55		54	53	
3	50	13		50	13	
*Depth of invasion*						
T1	1	6	*P*=0.001	2	5	*P*=0.001
T2	19	48		24	43	
T3	76	29		77	28	
T4	12	1		11	2	
*Lymph node*						
N0	50	73	*P*=0.001	58	65	*P*=0.001
N1	43	8		40	11	
N2	15	3		16	2	
*Distant metastasis*						
Negative	98	84	*P*=0.004	104	78	*P*=0.007
Positive	10	0		10	0	
*TNM stage*						
I	5	42	*P*=0.001	9	38	*P*=0.001
II	39	31		44	26	
III	54	11		51	14	
IV	10	0		10	0	

*P*<0.05, statistically significant.

### Correlation between Lgr5 and Ki-67 by Spearman’s correlation test

Ki-67 expression was positive in 59.4% (114/192) of the colorectal carcinomas and was found in the nuclei of cancer cells (Figure 1D). It was associated with tumor size (*P*=0.006), histological grade (*P*=0.001), invasive depth (*P*=0.001), metastasis in the regional lymph nodes (*P*=0.001), distant metastasis (*P*=0.007), pTNM stage (*P*=0.001), and prognosis (*P*=0.001). A positive correlation between Lgr5 and Ki-67 was found (r=0.446, *P*=0.001) (Table 2).

**Table 2:** Correlations between Lgr5 and Ki-67 expression in colorectal carcinoma

	**Lgr5 expression**		***P *value**	**r value**
	**Positive**	**Negative**		
*Ki-67 expression*				
Positive	85	29	0.001	0.446
Negative	23	55		

*P*<0.05, statistically significant.

### Survival analysis

**Figure 2:**
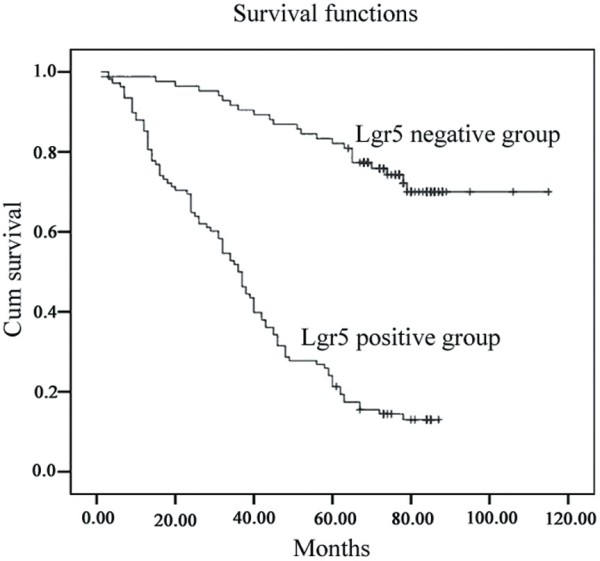
**Kaplan-Meier survival analysis by Lgr5 status (*n*=192).** The y-axis represents the percentage of patients, and the x-axis represents their survival in months. Lgr5-positive cases showed significantly shorter survival than Lgr5-negative cases by log-rank test. (*P*=0.001).

Follow-up data showed that the survival time of Lgr5-positive patients was significantly shorter (mean, 39.5 ± 4.8 months) than Lgr5-negative cases (mean, 95.6 ± 7.0 months) (log-rank test, *P* <0.001) (Figure 2). The overall 5-year survival rate of the Lgr5-negative group (83.3%) was better than that of the Lgr5-positive group (24.1%) (log-rank=80.27, *P*=0.001). Lgr5 expression was found to be an independent prognostic factor (*P*=0.001) by the Cox proportional hazard model. Other independent prognostic factors were age, histological grade, distant metastasis, TNM stage, and Ki-67 (Table 3).

**Table 3:** Cox regression analysis of prognostic factors in colorectal carcinoma

**Prognostic variables**	**B**	**SE**	**Wald value**	***P *value**	**HR**	**95% CI for HR**	
						**Lower**	**Upper**
Gender	0.051	0.205	0.063	0.802	1.053	0.705	1.572
Age (years)	0.262	0.130	4.037	0.045	1.299	1.006	1.677
Tumor size	0.031	0.187	0.027	0.869	1.031	0.715	1.487
Histological grade	0.541	0.179	9.184	0.002	1.718	1.211	2.438
Depth of invasion	0.235	0.204	1.325	0.250	1.264	0.848	1.885
Lymph node metastasis	−0.043	0.198	0.048	0.824	0.957	0.649	1.412
Distant metastasis	1.383	0.536	6.661	0.010	3.989	1.395	11.405
TNM stage	0.638	0.237	7.285	0.007	1.894	1.191	3.010
Lgr5 expression	1.018	0.274	13.842	0.001	2.768	1.619	4.732
Ki-67 expression	0.819	0.244	11.263	0.001	2.267	1.406	3.657

B, Partial regression coefficient; CI, Confidence interval; HR, Hazard ratio; SE, Standard error.

*P*<0.05, statistically significant.

### Hoechst low-staining cells identified in human colon carcinoma cell line Colo205

**Figure 3:**
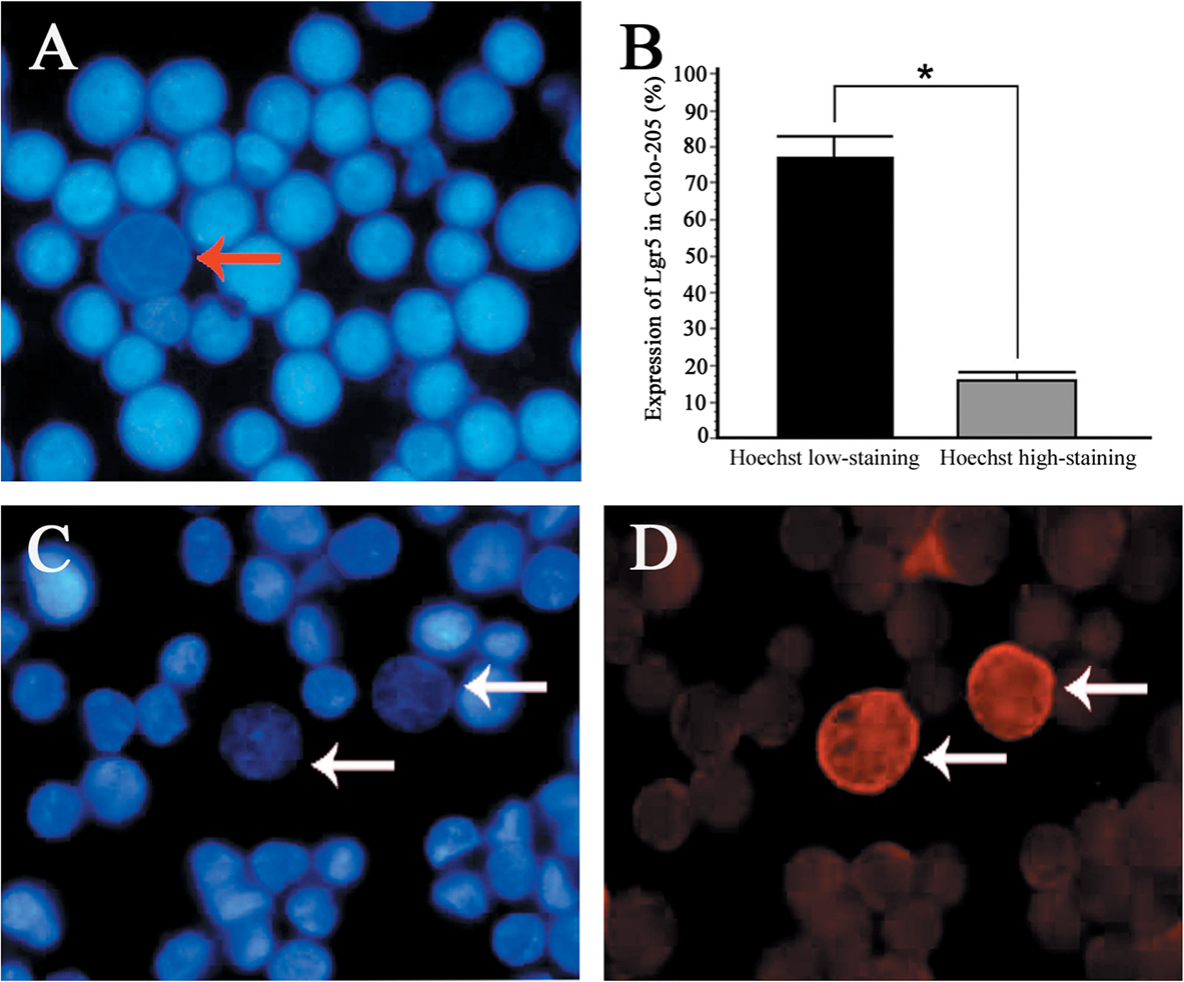
**(A) Results of Hoechst 33342 staining in fixed Colo205 colon cancer cells.** Red arrows indicate cells with low Hoechst 33342 staining. (**B, C, D**) Expression of Lgr5 in Colo205 colon cancer cells. The expression of Lgr5 was much higher in Hoechst 33342 low-staining cells (white arrows) than in high-staining cells (*P*=0.001). (A, C, and D: ×400).

To identify SP cells in established colon cancer cell lines, fluorescent microscopical analysis was used to detect cells that could effectively extrude the Hoechst 33342 dye. This assay revealed that in Colo205 cells, 7% of the cell population exhibited low Hoechst 33342 blue fluorescence, which identified them as SP cells (Figure 3A). The SP cells with low Hoechst 33342 staining were larger in size with pronounced round or oval nuclei, and possessed the morphological characteristics of stem-like cells. In Colo205 cell lines, the proportion of SP cells decreased to 0.38% following addition of the Hoechst 33342 dye transport inhibitor, verapamil.

### Expression of Lgr5 in Hoechst 33342 low-staining cells

Microscopical analysis showed that Lgr5 was stained strongly in the cytoplasm as well as the membrane. The expression of Lgr5 was obviously much stronger in Hoechst 33342 low-staining SP cells (76.84 ± 6.15%) than in Hoechst 33342 high-staining cells (15.87 ± 2.49%) (*P*=0.001) (Figure 3B, C, and D).

## Discussion

The CSC theory has become an intensely investigated topic, even though the origin of CSCs remains elusive and the isolation and identification of CSCs has not been achieved for many types of human tumor. CSCs are defined as a small side population of tumor cells with the ability to self-renew and potentially promote the formation of tumors [28-30]. Conventional cancer treatments indiscriminately kill proliferating cells and are always unsuccessful due to the survival of quiescent CSCs. Therefore, therapies could be designed to target cancer stem cells by inducing their differentiation or to eliminate them by inhibiting the maintenance of the stem-cell state [6,31]. CSCs can survive radiation therapy and chemotherapy and then return to a proliferative growth state, making them good targets for biomarker identification [15]. The cellular origin of cancer stem cells has not been clearly determined. GPCRs have been hypothesized to be closely associated with CSCs during tumorigenesis [13,32]. Lgr5, a member of the GPCR superfamily, is known as a stem cell marker in the small intestine and colon, as well as in hair follicles [11,33]. Lgr5 over-expression has been reported in a few cancers [10,12-15], including hepatocellular carcinoma, colorectal cancer, ovarian cancer, basal cell carcinoma, and esophageal adenocarcinoma. Recent studies suggested that Lgr5 may be involved in colorectal carcinogenesis as a target of Wnt signaling [8,9] and may be an ideal marker of colorectal CSCs [18,19]. In this study, Lgr5 was significantly overexpressed in the majority of CRCs (56.3%) compared with distal normal mucosa (25%). Increased expression of Lgr5 was significantly correlated with depth of invasion, lymph node metastasis, and distant metastasis. These results suggest that high expression levels of Lgr5 receptors are usually correlated with more malignant and metastatic tumors. Furthermore, Lgr5 was seen more frequently in advanced colorectal cancer. Positive expression of Lgr5 was found in 83.1% (54/65) of stage III cancer tissues and 100% (10/10) of stage IV cancer tissues. These results suggest that Lgr5 may play an important role in the development and progression of the tumor. It was also found that Lgr5 is closed related to the worst prognosis of colorectal carcinoma. In the present study, Lgr5 expression correlated with poor survival of colorectal cancer, indicating that Lgr5-positive cells may contain more CSCs. Therefore, in agreement with CSC theories, the residual CSCs may lead to metastasis and recurrence of colorectal cancer through multilineage differentiation and self-renewal after routine therapy [17,34]. Our study also showed that Lgr5 expression was positively correlated with Ki-67, a recognized nuclear antigen-specific marker of cellular proliferation, suggesting that Lgr5-positive cancer cells have higher proliferative activity.

Furthermore, we attempted to identify the Hoechst 33342 low-staining (SP) cancer stem-like cells in colorectal cancers using immunocytochemistry after selecting the cells that extruded Hoechst 33342. In our experiments, the SP cells were observed at a frequency of 7% in the Colo205 cell line, which was higher than reported by Kondo [2] and Patrawala *et al.*[35], and also proved the conclusion in the report by Telford *et al.*[36]. In order to better recognize the SP cells directly by microscopy, we first used immunofluorescence to observe and analyze the colon cancer cells expressing Lgr5 after Hoechst33342 staining. The results demonstrated that the expression of Lgr5 was obviously much stronger in the Hoechst 33342 low-staining SP cells than in the other cells. Therefore, it is suggested that the increased expression of Lgr5 may be a positive marker of SP cells in the Colo205 colon cancer cell line. Recently, many studies have focused on the identification of colon CSCs because of their potential for colorectal cancer treatment [37-39]. CD133 has recently been identified as a potential cancer stem cell marker [37,38]. However, controversy still remained because it had been reported that CD133 expression is not restricted to intestinal stem or cancer-initiating cells, and both CD133+ and CD133- metastatic colon cancer cells could initiate tumors [40]. It has been shown that CD133 expression is broadly distributed in colorectal cancer cells [41,42]. Thus, CD133 appears to be an inappropriate marker for the characterization of single colon CSCs [41]. Our results suggested that the expression of Lgr5 may correspond to the formation and function of Hoechst 33342 low-staining SP cells in colon cancer, and may differentiate these cells from other non-SP cells. Our results should be considered preliminary and require further confirmation by more in-depth functional studies both in cell lines and solid tumor samples.

## Conclusion

In conclusion, Lgr5 is highly expressed in colorectal cancer cells. The combined detection of Lgr5 and Ki-67 expression, to some extent, may reflect the biological behavior of colorectal cancer. Thus Lgr5 may be a potential new therapeutic target for the treatment of colorectal cancer patients, particularly those with advanced colorectal cancer. Additionally, Lgr5 appears to be a relevant candidate marker of colorectal CSCs. Our results are helpful for understanding the mechanism by which tumorigenesis is initiated, and for enabling targeted treatment of CSCs to prevent relapse or metastasis.

## Competing interests

The authors declare they have no competing interests.

## Authors’ contributions

XSW participated in the study design, carried out most of the experiments, performed the histological evaluation, and drafted the manuscript; HQX analyzed and interpreted the data, and carried out some of the experiments; LC participated in its design and gave final approval of the version to be published. All authors read and approved the final manuscript.
